# Residential and School Food Swamps and Overweight in Children and Adolescents: A Cross-Sectional Analysis in Urban Brazil

**DOI:** 10.3390/ijerph22081240

**Published:** 2025-08-08

**Authors:** Ingrid Werneck Linhares, Paula Martins Horta, Ariene Silva do Carmo, Luana Lara Rocha, Mariana Zogbi Jardim, Olivia Souza Honório, Larissa Loures Mendes

**Affiliations:** 1 Postgraduate Program in Health Sciences, Child and Adolescent Health, Universidade Federal de Minas Gerais, Belo Horizonte 30130-100, Brazil; ingridwe@ufmg.br (I.W.L.); zogbij@ufmg.br (M.Z.J.); 2 Department of Nutrition, Escola de Enfermagem, Universidade Federal de Minas Gerais, Belo Horizonte 30130-100, Brazil; pozinhamh@ufmg.br; 3 Department of Public Health, Universidade Federal de Minas Gerais, Belo Horizonte 30130-100, Brazil; ariene.carmo@mds.gov.br (A.S.d.C.); luanalararocha@ufmg.br (L.L.R.); 4 School of Nutrition, Centro Universitário Governador Ozanam Coelho, Ubá 36506-022, Brazil; olivia.honorio@aluno.ufop.edu.br

**Keywords:** food swamps, schools, overweight, children, adolescents

## Abstract

The community food environment, which encompasses residential and school neighborhoods, is an important determinant of overweight in children and adolescents. This study aimed to investigate the relationship between the co-occurrence of food swamps in residential and school environments and overweight status. This cross-sectional study included 2601 children and adolescents (aged 5–14 years) from 47 schools in a medium-sized municipality in Brazil. The outcome was overweight status, defined as body mass index for age exceeding the mean by at least one z-score. Food swamps in residential and school surroundings were defined as buffers of 250 m with four or more establishments selling ultra-processed foods. The prevalence of being overweight was 30.4%. Food swamps were present in 22.5% and 22% of the residential and school areas, respectively, and 16.2% of the participants were exposed to food swamps in both residential and school environments. Children and adolescents simultaneously exposed to food swamps in both residential and school areas had a higher likelihood of being overweight (odds ratio: 1.22; 95% confidence interval: 1.02–1.45). The simultaneous presence of food swamps in residential and school environments is associated with overweight in children and adolescents.

## 1. Introduction

Overweight and obesity are major global public health challenges affecting individuals across all ages and socioeconomic groups [1,2]. In Latin America and the Caribbean, more than 49 million children and adolescents are overweight [3]. Notably, being overweight during childhood and adolescence is associated with an increased risk of obesity in adulthood and the development of non-communicable diseases such as type 2 diabetes and hypertension [4].

The etiology of childhood and adolescent overweight is multifactorial, encompassing biological, behavioral, social, environmental, and even climate-related determinants [5]. Among behavioral factors, excessive consumption of ultra-processed foods stands out, as it has been extensively linked to weight gain during childhood and adolescence [6]. This eating pattern is directly related to the availability, accessibility, and continuous exposure to these products in spaces frequently visited by children and adolescents, such as homes and schools. Furthermore, unhealthy food choices are influenced by a complex set of factors including aggressive marketing strategies, food pricing, parental education, psychosocial stress, and broader socioeconomic conditions. These aspects contribute not only to current consumption patterns but also to long-term health trajectories into adulthood [7,8,9,10,11].

In this context, the food environment, defined as the interface between individuals and the food system [12], emerges as a key determinant of nutritional status. The community food environment, particularly in the surroundings of homes and schools, has been shown to influence children’s and adolescents’ food consumption patterns [13,14]. Evidence also suggests that early exposure to environments with limited healthy food options can shape dietary habits and preferences that persist over time, influencing the risk of chronic diseases later in life [15]. One way to characterize this environment is through the typology known as the “food swamp”, which describes areas with a high density of establishments selling predominantly unhealthy foods and beverages, such as ultra-processed foods [15,16,17].

Although several studies have explored associations between food outlet density and overweight among children and adolescents in residential [18,19,20,21] and school contexts [22,23], most evidence comes from high-income countries. Furthermore, to the best of our knowledge, no research has assessed the presence of food swamps in school and residential areas in relation to childhood overweightness, including an analysis of simultaneous exposure to both environments, especially in low- and middle-income countries. Exploring this interaction may be essential for a deeper understanding of the territorial determinants of overweight in children and adolescents.

Thus, this study aimed to assess the association between the simultaneous presence of food swamps in residential and school surroundings and overweight children and adolescents attending public schools in a medium-sized Brazilian municipality. We hypothesize that children and adolescents simultaneously exposed to food swamps in both residential and school environments have a higher likelihood of being overweight when compared to those exposed in only one or neither of these settings.

## 2. Materials and Methods

### 2.1. Study Design and Sample

This cross-sectional study was based on secondary data obtained through the Programa Saúde na Escola (PSE) between March and November 2019 in the municipality of Betim, Minas Gerais.

The PSE is an intersectoral initiative of the Ministries of Health and Education aimed at supporting the full development of students in public basic education schools through collaboration between the Primary Health Care System (Sistema Único de Saúde) and schools. Established in 2007, the PSE has evolved based on guidelines that integrate health promotion and prevention policies across 14 thematic areas, including the promotion of adequate and healthy eating and preventing obesity, with the inclusion of anthropometric assessments [24].

The sample included children and adolescents aged 5–14 years, enrolled in municipal public schools. Here, as defined by the World Health Organization (WHO) [25], children and adolescents were identified as individuals aged 5–9 years and 10 years or older, respectively.

Betim is located in the Metropolitan Region of Belo Horizonte, Minas Gerais, and is considered a medium-sized municipality with approximately 411,846 inhabitants. According to the Brazilian Institute of Geography and Statistics, the population density was 1197.01 inhabitants per square kilometer in 2022 [26].

### 2.2. Ethical Aspects

This study was conducted in accordance with the guidelines of the Declaration of Helsinki. This study was approved by the Research Ethics Committee of the Federal University of Minas Gerais and the Municipal Health Department of Betim (approval number: 4.656.224; CAAE: 41186020.7.0000.5149).

### 2.3. Children and Adolescents Database

The database comprised anthropometric records of students collected through PSE activities conducted in 2019. Microdata from these assessments were stored in an information system managed by the municipality. Of the 94 municipal public schools, 60 participated in the PSE, 56 conducted anthropometric assessments, and 47 were included in the analysis as they had complete weight and height data recorded in the system. For these 47 schools, data were available for 2654 individuals aged 5–14 years. The records included sociodemographic information (sex and age), anthropometric measurements (weight and height), date of birth, date of assessment, and residential address.

After excluding individuals residing outside the municipal boundaries (*n* = 31) and those with incomplete records (*n* = 22), the final sample consisted of 2601 children and adolescents.

### 2.4. Study Dependent Variable

Anthropometric assessments of children and adolescents included measurements of weight and height. Based on these data, the body mass index (BMI) (weight [kg]/height (m)^2^) for-age z-score was calculated using the WHO Anthro Plus software (version 1.0.4, World Health Organization, Geneva, Switzerland). When BMI for age exceeds the average by more than z-scores, the variable indicates overweight status [27,28]. This dichotomous variable was considered the dependent variable. Notably, health professionals working in health units have been trained to measure anthropometric data.

### 2.5. Study Explanatory Variables

The explanatory variables were food swamps in the vicinity of homes and/or schools.

Data on the food retail trade for 2019 were obtained from the State Department of Finance of Minas Gerais based on the National Classification of Economic Activities [29]. The following establishments were identified: snack bars, mini-markets, grocery stores, and candy shops.

The geographic coordinates (latitude and longitude) of residences, schools, and food retail establishments were obtained using their respective addresses via the Google Maps online service (https://www.google.com.br/maps?hl=pt-BR, accessed on 10 March 2021). The data were collected in the World Geodetic System 1984 geographic coordinate system and subsequently transformed into the Universal Transverse Mercator coordinate system, zone 23S, using the SIRGAS 2000 datum, with QGIS 2.10.1 software (https://qgis.org/pt_BR/site/, accessed on 4 November 2021).

Food swamps were identified following a methodology adapted from Hager et al. [30] and previously used in Brazil [31,32]. Additionally, the use of this method is justified by its prior testing in the Brazilian context, where food deserts were more prevalent in the lowest-income census tracts [33]. A food swamp was defined as four or more establishments primarily selling ultra-processed foods within a 250 m radius (Euclidean buffer) of the home or school. This distance corresponded to approximately 5 min of walking [34]. Home and school locations were used as buffer centroids.

### 2.6. Socioeconomic Characteristics of Residential and School Areas

Socioeconomic characteristics were assessed based on the per capita income of the census sectors of residence and school using data from the 2010 Demographic Census [35]. Income is expressed as the median and interquartile range. For reference, the minimum wage in 2010 was BRL 510.00.

### 2.7. Data Analysis

Descriptive analysis was performed using absolute and relative frequencies for categorical variables and median and interquartile range (IQR) (25th–75th percentiles) for continuous variables. Comparisons between groups were made using the Pearson Chi-square test (categorical variables) and the Mann–Whitney test (continuous variables).

Generalized estimating equation (GEE) models were used to assess the association between exposure to food swamps (residential and/or school surroundings) and being overweight. School was defined as the cluster unit, and the models were adjusted for sex, age, and per capita income in the census sector. Odds ratios (ORs) and 95% confidence intervals (CIs) were determined.

Additionally, GEE models were created with the addition of interaction terms to assess whether age group, sex, and income of the census tract (where the schools and households are located) modified the relationship between food swamps and excess weight.

Stata 17.0 software was used, and a significance level of 5% (*p* < 0.05) was adopted.

## 3. Results

The sample consisted of 2601 children and adolescents (53.0% male). Regarding age, 54.3% and 45.7% were children aged 5–9 years and 10–14 years, respectively. The overall prevalence of being overweight was 30.4%, which was significantly higher among adolescents (33.2%) than among children (28.1%) (*p* = 0.004) (Table 1). Regarding the food environment, 22.5%, 22.0%, and 16.2% of the participants lived in areas classified as food swamps, attended schools located in food swamps, and were simultaneously exposed to food swamps in both residential and school environments, respectively (Table 1).

The median per capita income of the census sectors in the locations of the schools and residences was BRL 439.30 (IQR: 395.4–523.6) and BRL 424.60 (IQR: 367.1–516.4), respectively. No statistically significant differences were observed in the per capita income of the sectors where the schools were located (BRL 439.30; IQR: 395.4–510.9 vs. BRL 432.00; 95% CI: 395.4–507.7, *p* = 0.117) and the residences (BRL 424.60; IQR: 368.5–521.0 vs. BRL 424.60; 95% CI: 363.1–509.8, *p* = 0.277) between the groups with and without overweight.

Multivariate analysis showed that simultaneous exposure to food swamps in both residential and school environments increased the likelihood of students being overweight by 22% (adjusted OR: 1.22; 95% CI: 1.02–1.45; *p* = 0.024). No significant associations were observed when exposure occurred only in the residential or school environments (Table 2).

In the analyses that tested whether individual and contextual variables modified the effect of the relationship between food swamps and excess weight, no significant interactions were found between swamps and age group (*p* = 0.811), sex (*p* = 0.123), income of the census tract where the school was located (*p* = 0.102), or household income (*p* = 0.642).

## 4. Discussion

This study examined the relationship between the coexistence of food swamps in both residential and school environments and overweight children and adolescents enrolled in public schools in Betim, Minas Gerais, Brazil. The results revealed a prevalence of being overweight (30.4%) in the study population, with a higher frequency among adolescents. Notably, simultaneous exposure to food swamps in both residential and school environments was significantly associated with overweight, independent of sex, age, or per capita income in the census sector.

Previous studies conducted in other countries have identified a positive association between the presence of food swamps in residential areas and increased consumption of unhealthy foods among adolescents [30], as well as higher obesity rates in adults [35]. A study conducted in adults in the United States found that the effect of food swamps on obesity was more substantial in regions with greater income inequality and where residents had lower mobility [36]. Systematic reviews have shown that the presence of establishments selling unhealthy foods in both residential and school areas, such as fast food chains and convenience stores, is associated with overweight and obesity in children and adolescents [18,19,20,21,22,23].

Other studies conducted in different Brazilian cities found a high concentration of establishments selling ultra-processed foods around schools, identifying a high frequency of schools located in food swamps [31,32,37,38,39,40]. These data corroborate the findings of Betim and emphasize the need for regulatory actions focused on the school food environment, prioritizing the sale of food around these spaces.

The results of this study demonstrated that the presence of food swamps in only one location (either around schools or residential areas) was not associated with overweight. However, simultaneous exposure correlated with a higher risk of overweight among students, suggesting that the overlap of unhealthy food environments in different areas of daily circulation may increase the risk of becoming overweight. These findings support the hypothesis that the combination of home and school environments within food swamps intensifies access to ultra-processed foods, favoring inadequate dietary patterns from childhood. Simultaneous exposure to food swamps reflects the daily life in territories marked by social vulnerability and the precariousness of public policies related to urban planning and food environment regulation.

The findings of the present study highlight the urgency of intersectoral and multicomponent interventions that include regulatory measures, such as zoning laws restricting access to unhealthy food establishments in specific areas like schools, that encourage healthy food retailers to establish themselves in neighborhoods—particularly in more vulnerable ones—and that promote educational strategies to foster adequate and healthy eating among children, adolescents, and their families [30,36]. An 8-month intervention conducted in the United States aimed at increasing the availability and selection of healthy foods through nutrition education and promotion, utilizing point-of-sale materials such as posters and leaflets in stores, as well as interactive sessions, such as taste tests and cooking demonstrations, was associated with reductions in BMI among youth [41].

Despite the relevance of these results, this study has some limitations. The primary concern is its cross-sectional design, which hinders the establishment of causal relationships between exposure and outcomes. Furthermore, the study is based on socioeconomic data from the 2010 census, which may not accurately reflect the current reality of the territories. Another limitation is the lack of information on the informal food trade, which is common in urban areas of Brazil and may have led to an underestimation of actual exposure to food swamps. Finally, behavioral variables (such as eating habits and physical activity levels) were not analyzed, which may have influenced the interpretation of the results. Future studies should include these variables because socioecological theoretical models suggest that behavioral factors such as diet and physical activity mediate the relationship between the food environment and childhood excess weight [42,43].

Despite these limitations, this study makes significant contributions to the field. To our knowledge, this is the first study to analyze simultaneous exposure to food swamps in both residential and school environments, as well as their association with overweight children and adolescents. By filling this gap, this study contributes to expanding our understanding of how the community food environment can act as a social and territorial determinant of overweight in childhood and adolescence. The results highlight the structuring role of territory in the production of food and nutritional inequalities.

## 5. Conclusions

This study identified that one in three children and adolescents enrolled in public schools in Betim (MG) were overweight, and that simultaneous exposure to food swamps in both residential and school environments was significantly associated with this condition. These findings reinforce the community food environment’s role as a significant determinant of health inequities among children and adolescents in urban settings.

The present findings expand the scientific evidence on the need to regulate food sales around schools, which is currently underrepresented in most Brazilian legislation. Furthermore, they emphasize the importance of intersectoral public policies combining territorial regulation strategies with educational actions to promote adequate and healthy eating, especially within the scope of PSE and Basic Health Units.

Given the persistence and worsening of overweight in vulnerable populations, the urban food environment must be readily recognized as a central component of health, education, and urban planning policies. Integrated and intersectoral measures are essential for transforming the territory into a space that promotes health and equity in future generations.

## Figures and Tables

**Table 1:** Characterization of children and adolescents included in the study, categorized by the presence of overweight. Betim, Minas Gerais, Brazil (*n* = 2601, 2019).

Variables	Total (%)	Body Mass Index by Age		*p*-Value *
		Without Overweight (%)	With Overweight (%)	
Sex				0.315
Female	47.0	70.6	29.4	
Male	53.0	68.7	31.3	
Age group				0.004
5–9 years	54.3	71.9	28.1	
10–14 years	45.7	66.8	33.2	
Food Swamps				0.089
None	39.18	70.56	29.44	
Only in the residential area	22.53	71.84	28.16	
Only in the school area	22.03	69.28	30.72	
Simultaneously in both residential and school areas	16.26	64.78	35.22	

* Pearson’s Chi-Square Test.

**Table 2:** Association between food swamps in the residential and school environments and overweight among children and adolescents from municipal schools participating in the Programa Saúde na Escola (PSE). Betim, Minas Gerais, Brazil (*n* = 2601, 2019).

Variables	Overweight					
	Unadjusted OR	CI 95%	*p*-Value *	Adjusted OR	CI 95%	*p*-Value *
Food Swamps						
None	Reference	-	-	Reference	-	-
Presence in the residential area	0.94	(0.80–1.11)	0.533	0.93	(0.79–1.10)	0.425
Presence in the school area	1.03	(0.86–1.22)	0.723	1.10	(0.93–1.31)	0.283
Simultaneous presence in residential and school areas	1.16	(0.97–1.39)	0.095	1.22	(1.02–1.45)	0.024

Note: OR: Odds Ratio; CI: Confidence Interval. * Generalized Estimating Equations model adjusted for sex, age, and per capita income in the census sector.

## Data Availability

The data are available upon request from the municipality of Betim, Brazil.

## Abbreviations

The following abbreviations are used in this manuscript:

NCDs	Non-communicable diseases
PSE	Programa Saúde na Escola
SUS	Sistema Único de Saúde
WHO	World Health Organization
IBGE	Brazilian Institute of Geography and Statistics
BMI	Body Mass Index
UTM	Universal Transverse Mercator
IQR	Interquartile range
GEE	Generalized Estimating Equations
CI	Confidence intervals
OR	Odds ratios
